# Elements of Human Anatomy; General, Descriptive, and Practical

**Published:** 1867-07

**Authors:** 


Elements of Human Anatomy; General, Descriptive, and Practical. By T. G. Richardson, M.D., Prof. of Anatomy in the Medical Department of the University of Louisania. Second Edition. Carefully Revised, and Illustrated by nearly 300 engravings. Philadelphia: J. B. Lippincott & Co. 1867.

This is a full-sized octavo volume of 671 pages; published in excellent style. Its illustrations are well executed, and, as a whole, it constitutes one of the best text-books on anatomy that we have seen.

For sale by S. C. Griggs & Co., 41 Lake Street, Chicago. Price $6.00.

